# Does Explicit Expectation Really Affect Preparation?

**DOI:** 10.3389/fpsyg.2012.00378

**Published:** 2012-10-01

**Authors:** Valentin J. Umbach, Sabine Schwager, Peter A. Frensch, Robert Gaschler

**Affiliations:** ^1^Department of Psychology, Humboldt-Universität zu BerlinBerlin, Germany; ^2^Berlin Research Center Image Knowledge Gestaltung, Humboldt-Universität zu BerlinBerlin, Germany

**Keywords:** explicit expectation, action control, anticipation, preparation, task goals

## Abstract

Expectation enables preparation for an upcoming event and supports performance if the anticipated situation occurs, as manifested in behavioral effects (e.g., decreased RT). However, demonstrating coincidence between expectation and preparation is not sufficient for attributing a causal role to the former. The content of explicit expectation may simply reflect the present preparation state. We targeted this issue by experimentally teasing apart demands for preparation and explicit expectations. Expectations often originate from our experience: we expect that events occurring with a high frequency in the past are more likely to occur again. In addition to expectation, other task demands can feed into action preparation. In four experiments, frequency-based expectation was pitted against a selective response deadline. In a three-choice reaction time task, participants responded to stimuli that appeared with varying frequency (60, 30, 10%). Trial-by-trial stimulus expectations were either captured via verbal predictions or induced by visual cues. Predictions as well as response times quickly conformed to the variation in stimulus frequency. After two (of five) experimental blocks we forced participants by selective time pressure to respond faster to a less frequent stimulus. Therefore, participants had to prepare for one stimulus (medium frequency) while often explicitly expecting a different one (high frequency). Response times for the less frequent stimulus decreased immediately, while explicit expectations continued to indicate the (unchanged) presentation frequencies. Explicit expectations were thus not just reflecting preparation. In fact, participants responded faster when the stimulus matched the trial-wise expectations, even when task demands discouraged their use. In conclusion, we argue that explicit expectation feeds into preparatory processes instead of being a mere by-product.

## Introduction

“You have to expect things of yourself before you can do them,” as stated by basketball legend Michael Jordan (http://www.biography.com/people/michael-jordan-9358066). Expectation is elemental in many types of behavior. It allows us to predict and prepare for an upcoming event. It can be implicit, as when we are not aware of it, or explicit. Here we focus on explicit expectations pertaining to an upcoming stimulus. These expectations can be either based on experienced stimulus frequency (made explicit through verbal predictions) or based on cues providing advance information.

Many researchers stress the role of expectation in controlling our behavior (e.g., Kunde et al., 2007; Duthoo et al., 2012). The quote above is just one example of how we take for granted that expectations influence how we go about a task. However, there are prominent findings on action control, which demonstrate that the colloquial notion of expectations influencing preparation needs empirical support. For instance, a recent brain imaging study by Soon et al. (2008) found brain activity reflecting the preparation for a free choice up to 10 s before it entered awareness (mirroring the classic “free will” experiment by Libet et al., 1983). Conscious intention might thus only be an epiphenomenon of preparatory processes in the brain (but see Trevena and Miller, 2010, for opposing evidence). Similarly, when asking someone to verbalize their expectation (about a future event that they will have to respond to) it is unclear whether the verbalized expectation simply *reflects* a preparatory state or whether it can in addition influence task processing. According to the latter view, an explicit expectation (which might be rooted in preparatory processes to some extent) feeds back into task processing. For instance, preparatory processes might be slightly stronger for one vs. another stimulus at the moment an explicit expectation is generated. The explicit expectation might feature just one of the stimuli and preparation for this option might be amplified in a winner-takes-it-all manner, because an explicit expectation had to be generated.

While the notion of expectation as a distinct construct has served as an example for redundant theorizing by critics of early cognitive psychology (e.g., Skinner, 1950) it has gained considerable support through cognitive modeling, where prediction error terms are at the core of many learning models (e.g., Sutton and Barto, 1981), as well as through the discovery of neural correlates (e.g., Schultz et al., 1997). According to Gallistel (2005) expectations have a causal role in human behavior in many economic theories and are the driving force of fast adaptation in animals to changed reinforcement schedules. The concept of expectation is discussed under various labels such as anticipation (e.g., Kunde et al., 2007), expectancy (e.g., Perruchet et al., 2006), and prediction (e.g., Sutton and Barto, 1981). Expectation encompasses both the act of looking forward as well as the thing looked forward to. In the current study, we refer to expectation as the explicit verbal prediction (or descriptive cue) of an upcoming stimulus in a sequential choice task.

In the current study, we wish to put the notion that explicit expectations have a causal role in preparation to the test. As in the work by Soon et al. (2008) we use a broad concept of preparation, encompassing any process, or state of the cognitive system that promotes the (speedy and accurate) execution of a certain action. This can take place anywhere along the cognitive processing chain, from attentional preparation (perception) to response selection (decision) to motor preparation (action). Faster responding has been shown if orientation of attention is possible in advance and facilitates perception (e.g., Posner and Petersen, 1990). On the other hand, processes of response selection and execution also benefit from preparation based on available advance information (e.g., Rosenbaum and Kornblum, 1982), which then results in faster responding. Wherever the facilitation takes place, a prepared action should be executed faster (as measured by RT). Here, we talk about *match effects* when comparing cases in which the required response matches the expectation, vs. cases in which it does not.

Expectations often originate from our experience: we expect that events occurring with a high frequency in the past are more likely to occur again in the future (e.g., Fitts et al., 1963). According to information theory (Shannon, 1948), information gain is low if an event encountered frequently before re-occurs. On the one hand, in this case little can be learned. On the other hand, the occurrence of the expected event usually boosts performance, whereas unexpected events can cause cognitive conflict and impair performance (e.g., Bernstein and Reese, 1965; Posner and Snyder, 1975). In line with the view that explicit expectation can feed back into action preparation, Miller and Anbar (1981) have suggested two routes for the impact of event frequency on action preparation: directly by strengthening S-R associations and indirectly by subjective expectations.

However, in many task situations explicit expectations and other aspects of task preparation favor the same behavior. This renders it difficult to demonstrate that explicit expectation is influencing task processing above and beyond these other aspects. For instance, a frequent S-R connection might be favored both by the high strength of the S-R association as by an explicit expectation, but it is difficult to demonstrate that the latter is actually feeding back into preparatory processes in such a situation. Therefore, we developed a paradigm in which participants can be made to expect one event (by event frequency) while another task demand (severe time constraint on a stimulus which is not the most frequent one) at the same time requires that they are preparing for a different event. If explicit expectations have an effect on task processing in a situation in which one would be better off preparing for a different event than the one expected, this would considerably strengthen the view that explicit expectations are feeding back into preparatory processes. This approach borrows its rationale from Perlman and Tzelgov (2006) who suggested scrutinizing effects that are *not* adaptive. Often, cognitive psychology builds on concepts that lend their credibility to adverse performance effects. If the effect of interest disturbs efficient performance, it is hard to explain it away. In their case, the concept of implicit learning (as distinct from controlled learning processes that in some cases might run in parallel) could be considerably supported by showing that implicit learning takes place even when it hampers performance – more learning led to worse performance. Similarly, our notion of explicit expectation as a distinct source of task processing could be backed by demonstrating dysfunctional performance effects.

In line with our perspective, a recent study by Duthoo et al. (2012) points toward the use of expectation even when it is invalid. We want to extend this finding. If, for example, people expect an event they know is very unlikely to occur, are they still preparing for it? Finding performance gains in such a case (if the unlikely event does occur) would suggest a functional role of expectation (being translated into preparation), despite the largely dysfunctional effects. As a stronger test for the impact of explicit expectation on preparation we introduced a conflicting task demand promoting the preparation of an option different from the one expected. Preparation in terms of “response readiness” (Rosenbaum and Kornblum, 1982) should be susceptible to other influences besides advance information or stimulus expectation. For example, the reinforcement of a certain response should increase its preparation state even if expectation based on past experience or situational cues favors a different response. Significant match effects in this case would suggest an influence of explicit expectation even when it is maladaptive. On the other hand, following the view of conscious intention as epiphenomenon of unconscious determinants of behavior (Libet et al., 1983; Soon et al., 2008), explicit expectations in our study should change in line with changes in preparation. If explicit expectation is merely reflecting rather than influencing task preparation, then explicit expectation should change when task preparation is experimentally changed. There is evidence, however, that subjective expectations can deviate from action preparation based on priming or associative learning (Perruchet et al., 2006). If explicit expectation is assumed to have a function in cognitive processing (as opposed to being a mere by-product) it should not be altered by a task demand that selectively manipulates preparation.

In addition to past experience, expectation can also be based on situational cues. The distinction between these two sources of expectation has been largely overlooked in research on expectation effects (but see Acosta, 1982). Results from our lab (Kemper et al., 2012) point to significant differences: self-generated predictions are accompanied by a distinctive expectation state visible in the contingent negative variation of the electroencephalogram and have a stronger effect on sensoric potentials compared to external cues, resulting in larger behavioral effects. In order to target the role of explicit expectations in preparation on a broad basis, we used both types of explicit expectations in the current study.

## Materials and Methods

In a series of four experiments, we used a three-choice reaction time task. Stimuli were displayed with different frequencies, with one stimulus being presented in 60% of all trials, another one in 30%, and the last in 10% of all trials. Participants responded to each stimulus by pressing one of three keys. As a measure of trial-wise subjective expectation we asked participants to verbally predict the upcoming stimulus on each trial (Experiment 1: *verbal predictions*). To control for effects of this verbalization procedure, we ran a variant where no predictions were required (Experiment 2: *no predictions*). In two additional experiments, we replaced the self-generated predictions with external cues indicating the upcoming stimulus. Cues were either not predictive of the subsequent stimulus presentation (Experiment 3: *non-informative cues*), or they correctly indicated the upcoming stimulus on 80% of all trials (Experiment 4: *informative cues*). In order to test for effects of explicit expectation when it is not perfectly in line with other demands for task preparation, we introduced a response deadline for the medium frequency stimulus toward the second half of all experiments.

### Experiment 1: Verbal predictions

Responses to the more frequent stimuli should generally be faster because of stronger S-R associations and because they are expected more often (Miller and Anbar, 1981). Subjective predictions (in Experiment 1) should also reflect this frequency pattern, with participants more often predicting the more frequent stimuli. A common phenomenon in this context is the tendency of people to match their predictions to the observed probabilities, resulting in fewer correct predictions compared to an optimal strategy (i.e., always predict the most frequent event). This phenomenon has been described as probability matching (e.g., Gaissmaier and Schooler, 2008). Participants should display the same tendency in our task if they really try to predict the upcoming stimulus. Therefore, finding a frequency effect in explicit expectations provides a manipulation check to ensure that participants are in fact correctly performing the task of verbalizing their expectations in our experiment. While actual stimulus presentation was unrelated to these subjective predictions, responses should be faster after (coincidental) correct predictions if people use their predictions to prepare for task execution.

Faster responses to correctly predicted stimuli (match effects) would point toward a mandatory use of subjective expectation in action preparation. Since there is no relation between participants’ predictions and the actual stimulus they have to respond to, there is no reliable gain for them in following their predictions. This holds in particular for predictions of the two less frequent stimuli. To challenge the assumption of a mandatory use of explicit expectations even further, we introduced an additional task demand with the goal of diverting preparatory processes away from the response to the expected stimulus. After two of five experimental blocks participants were instructed to give particularly fast responses to occurrences of the medium frequency stimulus (30%). Slow responses on these trials were punished by presenting an unpleasant noise which acted as a negative reinforcement. This additional task demand was therefore at odds with the pattern set up by the stimulus frequencies. While stimulus frequency and subjective expectations should lead to faster responses for the most frequent stimulus, the additional task goal (avoid the unpleasant noise) should lead to a stronger preparation for the medium frequency stimulus. It makes preparation on the basis of frequency expectations less useful because preparing for the predicted response may result in hearing the aversive sound in some cases (i.e., when the frequent stimulus is predicted and prepared and the medium frequent stimulus occurs and is responded to too slowly). Still finding match effects under these conditions would be further evidence for the mandatory use of explicit expectation in preparing for an upcoming task. To the extent participants are able to adjust their preparation to the requirements of the actual task one could expect reduced expectation match effects in blocks three to five: participants should rely less on their stimulus predictions if the medium frequent response is reinforced.

Match effects (faster responses following correct predictions) are in line with our idea that people use their explicit subjective expectations in action preparation. However, there is the possibility that these expectations are simply a by-product of preparation without functional use. In this case, participants should adjust their predictions in line with the changes in action preparation once the additional task demand is established. If participants in fact prepare to respond to the medium frequency stimulus, and if their stimulus expectations are inseparably linked to this preparation (as in “reading out” an internal preparation state determined by the strength of specific S-R associations), this should be reflected in their prediction frequencies. In this case, match effects might not be reduced (see above), as both preparation and prediction would follow the altered task demands. If, on the other hand, people generate expectations independently of action preparation that is fueled by a second task demand, the frequency pattern should remain intact in their subjective predictions.

### Experiment 2: No predictions

In Experiment 1 verbal predictions were required before each stimulus occurrence resulting in a dual-task like situation: to generate verbal predictions and to perform the manual choice reaction task. This could have resulted in different processing of the choice task as compared to solely producing choice reactions. In order to verify the results found for frequency and, particularly, the effect of selective reinforcement of the medium frequent stimulus, we repeated the experiment without verbal predictions.

### Experiment 3: Non-informative cues

Expectation effects are most often investigated by using external advance information (provided by cues, e.g., Posner and Snyder, 1975; Miller and Anbar, 1981; Mattler, 2004). It has been shown, however, that expectations induced by cues affect performance differently from predictions generated by participants themselves (Kemper et al., 2012). Against this background we repeated Experiment 1 and replaced verbal predictions with visual, non-verbal cues that announced one of the three stimuli in advance before the imperative stimulus was presented. The probability of match was kept at approximately the same level as in the prediction experiment by presenting the cues with the same frequencies as the stimuli (10, 30, and 60%) but randomized independently of stimulus presentation. The general effect of stimulus frequency should be similar to the previous experiments, as well as the impact of the selective response deadline. In line with previous studies (Acosta, 1982; Kemper et al., 2012) we expect a smaller match effect with cues than with predictions.

### Experiment 4: Informative cues

We conducted Experiment 4 for two reasons. First, the use of non-informative cues is quite atypical for investigating expectation effects by the help of external advance information. Usually, cueing effects on preparation appear only with highly reliable cues (e.g., Alpay et al., 2009; Scheibe et al., 2009). The reason for finding an effect under such unfavorable conditions as in Experiment 3 might lie in feature overlap between cue and stimulus. Second, we wanted to explore an idea that could explain the difference in effectiveness between explicit expectations generated by the individual or provided by external advance information. As the overall real validity of predictions (Experiment 1) and cues (Experiment 3) was comparable the difference might in fact go back to the degree to which participants rely on their expectation, depending on its source. One possible mechanism could be that participants weight self-generated predictions stronger and that external information has to be of a much higher validity to be included into controlled action preparation, or, alternatively, predictions and cues differ in subjective usefulness.

Therefore, in Experiment 4 we increased the probability of match between cue and stimulus feature to 80%. Under these conditions a much larger effect of expectation match than in Experiment 3 should be observable. We expect comparable effects of stimulus frequency as in the previous experiments, as well as an effect of selectively reinforcing the medium frequent stimulus by use of a deadline.

#### Participants

One hundred five undergraduate students of psychology and other fields (74 women, mean age = 24.9 years) participated in individual sessions lasting approximately 90 min (Experiments 1 and 2) or 60 min (Experiments 3 and 4). Participants either received partial course credit or were paid 8–12 euros for their time. They provided written informed consent, particularly to the exposure to aversive sounds.

#### Design and procedure

**Figure 1:**
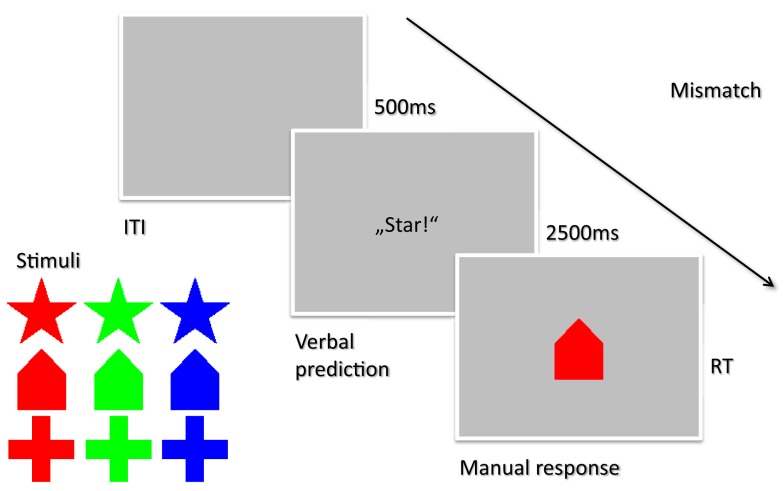
**Task used in Experiment 1**. On each trial, participants had to verbalize their prediction for the upcoming stimulus (in this case “star”). After 2500 ms the stimulus appeared on screen (in this case a house, signifying a mismatch) and participants had manually respond by pressing one of three keys. The next trial started 500 ms after the response. For any given participant, only one of the two stimulus features (shape, color) was relevant throughout the task (in this case, both predictions and responses pertained to the shape of a stimulus).

In all of the experiments reported here, we used a three-choice reaction time task. Three different shapes served as stimuli – star, house, and cross – that were presented in one of three colors, red, green, or blue. Each stimulus could be named by a monosyllabic word in order to provide for approximately equal verbalization times (for Experiment 1; German “Stern,” “Haus,” “Kreuz,” or “rot,” “grün,” “blau”). Stimuli were displayed centrally on a 17′′ CRT computer monitor with a light gray background and occupied approximately 2.2 cm in width and height (corresponding to a visual angle of about 6.4° at a viewing distance of 60 cm). Three keys (V, B, and N) on a standard Windows keyboard were mapped by instruction either to the three shapes or the three colors, with the relevant feature varying between participants. The task and stimuli are shown in Figure 1.

Frequency of the three possible shapes or colors, respectively, was predetermined in the stimulus set to yield three frequency classes, frequent (60%), medium (30%), and infrequent (10%) for the relevant stimulus feature. Occurrence of the irrelevant feature was equally distributed and co-occurrence was balanced across features. Half of the participants used shapes as relevant feature for predictions and response selection and the others used color. The irrelevant feature was not used in this task.

Participants completed five blocks of 120 trials for a total of 600 trials. The frequent stimulus occurred in 72 trials, the medium frequent in 32 trials, and the infrequent stimulus in 12 trials per block. After the first two experimental blocks the additional task demand was introduced. Participants were informed that their reactions to the medium frequency stimulus (which was simply described by its label) had to be extra fast if they wanted to avoid the annoying sound on their headphones. This aversive auditory stimulus, a white noise burst of about 75 dB, had been demonstrated to participants at the beginning of the session before they gave their consent to the procedure. The response deadline for the medium frequency stimulus was individually determined at the median reaction time for the frequent stimulus in the preceding Block 2 and kept constant over the remaining three blocks. If participants exceeded this deadline on any given trial with the medium frequency stimulus, the aversive sound was immediately presented on their headphones and ended 500 ms after their (late) reaction.

At the end of the session participants were asked to estimate the frequency of the relevant stimulus feature.

### Experiment 1: Verbal predictions

On every trial, participants were asked for their subjective expectation regarding the upcoming stimulus. According to the relevant stimulus feature, the prompt “Farbe?” or “Form?” (German for color or shape) were displayed on the screen. Participants then had 2500 ms to verbalize their expectation. If voice onset was registered more than 1500 ms after the onset of the prompt, participants were reminded to speak as soon as the prompt is shown on the next trial. In addition, participants were randomly reminded in 10% of all trials to speak loudly and clearly. After this expectation interval (2500 ms after the prompt onset) the stimulus was shown and participants had to press the corresponding key on the keyboard. The following trial started 500 ms after the response. The experimental blocks were preceded by three practice blocks of 18 trials each in which manual responses and verbal expectations were first trained separately and then combined. Frequency and combinations of relevant and irrelevant stimulus feature were equally distributed in the practice blocks.

Verbal expectations were captured with a microphone headset and identified using a real-time speech recognition program implemented in Matlab (Donkin et al., 2009). At the beginning of the experimental session, the software was trained to the individual voice with the participant repeating the words in the response set 10 times. This was followed immediately by an accuracy check with 10 additional exemplars per word. If recognition accuracy was below 95% (i.e., more than one misidentification) the original training was restarted, otherwise the additional exemplars were added to the pool of training exemplars and the experiment commenced. Recognition accuracy was tested again at the end of the session.

### Experiment 2: No predictions

The task was the same as in Experiment 1, with the only difference that participants were not instructed to generate verbal predictions at the beginning of each trial. Instead of the prompts used in Experiment 1 a fixation dot was displayed for 2500 ms to keep the timing equivalent to Experiment 1.

### Experiment 3: Non-informative cues

Again, the task was largely the same as in Experiment 1. Instead of prompting participants to verbalize their subjective expectations on each trial symbolic cues were presented predicting the upcoming stimulus. These cues were similar to the imperative stimuli but only varied in the relevant feature: if a participant had to respond to the shape of a (colored) stimulus the cues consisted of black shapes, if color was the relevant feature colored circles were used as cues. Participants did not have to verbalize the cues. Cues were displayed 1000 ms after the last response and remained visible for 1000 ms followed by a blank screen for another 1000 ms, after which the imperative stimulus appeared. Thus, the response-stimulus interval was the same as in the other experiments (3000 ms) and the timing of the cues was similar to the verbal predictions in Experiment 1. Importantly, cue presentation was randomized independently and was not related to the subsequent stimulus presentation. Therefore cues exhibited the same low overall validity as the predictions in Experiment 1: on only 46% of all trials was a cue followed by the corresponding stimulus (60% for the frequent stimulus, 30% for the medium, and 10% for the infrequent stimulus).

### Experiment 4: Informative cues

The task was the same as in Experiment 3, except that the validity of cues was 80% for all frequencies. Thus, in 80% of all trials a cue was followed by the corresponding stimulus.

## Results

### Experiment 1: Verbal predictions

Three participants were excluded for producing too many false responses (>10%), another two participants were excluded because of problems with the speech recognition software (<75% accuracy in the post-experiment test). Data of the remaining 19 participants were analyzed. For the following analyses all trials were recruited, including those with immediate stimulus repetitions. The proportion of stimulus repetitions naturally were related to stimulus frequency, with 60% repetition trials for the frequent stimulus, and 29 and 9% respectively for the medium and infrequent stimuli. All results reported here remain unaltered if stimulus repetitions, i.e., 46% of all trials, are excluded. RT analyses are based on correct responses only, excluding error trials. The response deadline, representing the median reaction time for the frequent stimulus in Block 2, was on average set at 424 ms (SD = 76 ms), with individual participants ranging between 303 and 633 ms. In 28% of the trials with the reinforced stimulus, participants passed this deadline and were consequently exposed to the aversive sound (32% in Block 3, 24% in Block 4, 27% in Block 5).

Our experiments, except Experiment 2 with no predictions, included three within-subjects factors: *match* (testing the effectiveness of explicit expectation), *block* (mirroring the effect of training and, more importantly, of the introduction of the response deadline from block 2 to block 3), and *frequency*. A three-factorial repeated measures ANOVA could not be run as participants did not contribute enough data points to one of the cells (match trials for the infrequent stimulus occurred too rarely to get reliable medians per block). Therefore, three two-way ANOVAs were run over the response times and error rates of all experiments: one with *frequency* and *block* to examine the general effect of selectively reinforcing the medium frequent response, one with *match* and *frequency* to look for a potential dependency of the size of expectation effects on experienced stimulus frequency, and one with *match* and *block* to examine the interaction of expectation and the deadline manipulation. In the context of a Bonferroni correction we divided the critical significance level (alpha = 0.05) by three in order to account for repeated tests on one and the same data set.

**Figure 2:**
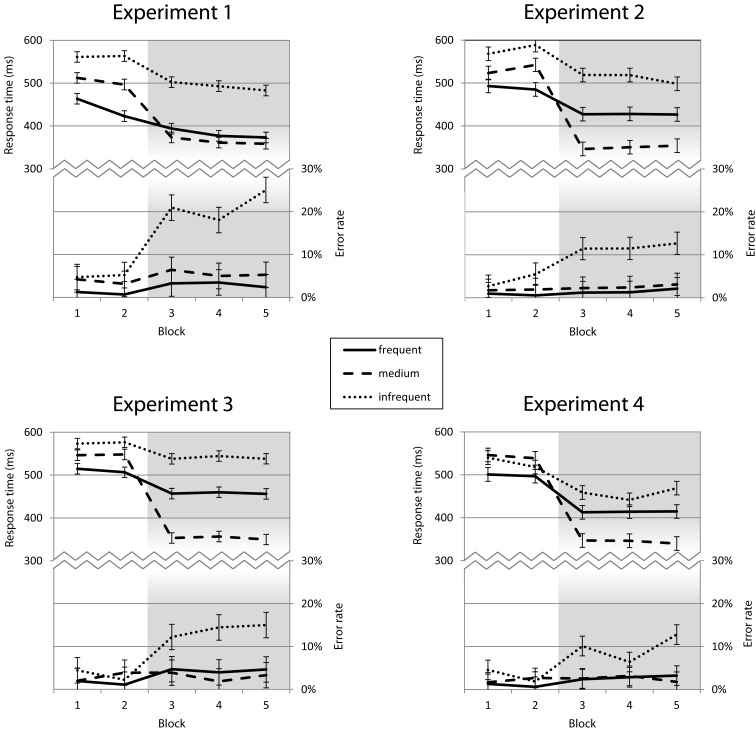
**Response times and error rates for Experiments 1–4**. Response times (on the top of each panel) exhibit an effect of stimulus frequency (with the frequent stimulus, marked by the solid line, leading to faster responses than the medium and infrequent stimuli) in the first two blocks, as well as an effect of the additional task goal starting in the third block (with the reinforced medium stimulus getting faster responses). The same pattern is visible in conditions with verbal predictions (Experiment 1) and without (Experiment 2) and also with low and high validity non-verbal cues (Experiments 3 and 4).

Before the introduction of the response deadline, RTs and errors followed stimulus frequency. The infrequent stimulus led to the slowest and most error prone reactions and the responses to frequent stimuli were the fastest and most accurate. The medium frequency stimuli lay in between. With the response deadline, in the last three blocks, responses to the medium frequency (reinforced) stimulus became faster than responses to the more frequent stimulus, while response times for all stimuli decreased. A two-way repeated measures ANOVA with the factors *frequency* and *block* revealed main effects for both *frequency*, *F*(2, 36) = 81.63, *p* < 0.001, and *block*, *F*(4, 72) = 82.27, *p* < 0.001, as well as an interaction, *F*(8, 144) = 15.91, *p* < 0.001. Importantly, the selective speedup of responses to the medium frequent stimulus was not achieved at the expense of a higher error rate for the frequent stimulus (see Figure 2, top left). The same effects as in RT were found in the error rates (all *p* < 0.001).

**Figure 3:**
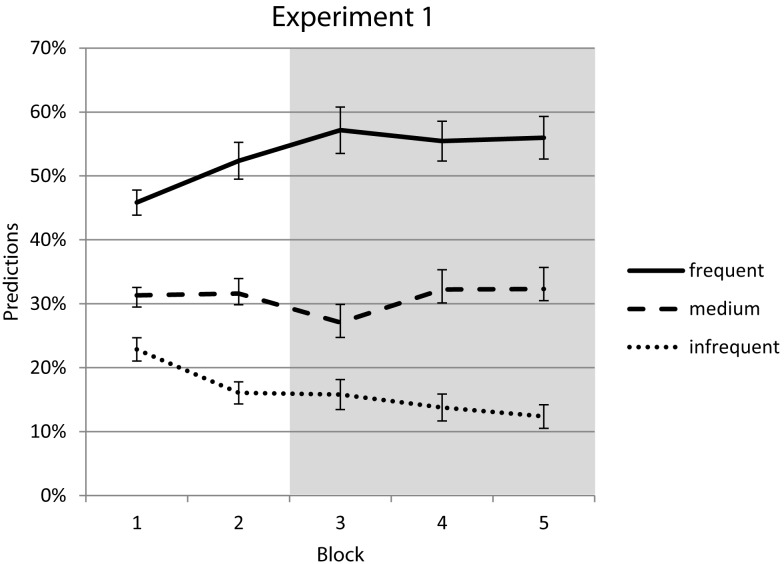
**Predictions in Experiment 1 already reflect the varying stimulus frequencies in the first block and approach the actual values (60, 30, and 10%) over the course of the experiment**. The additional task goal introduced in the third block does not change this pattern.

Verbal predictions already reflected the frequency differences in the first block and approached the actual values over the course of the experiment. Importantly, this pattern was not altered with the introduction of the response deadline in the third block (see Figure 3). Therefore, participants continued to expect the most frequent stimulus most often but reacted fastest to the medium frequency stimulus. The three different stimuli were predicted in the order of their frequency of occurrence (most often the most frequent stimulus, less often the medium frequent stimulus, and least often the rare stimulus). This rank order of prediction frequencies stayed the same over the experiment, so that prediction behavior was highly correlated over blocks (correlation of ranks between successive blocks: τ = 0.74, 0.79, 0.92, and 0.83, all *p* < 0.001), regardless of the changed pattern in choice performance.

**Figure 4:**
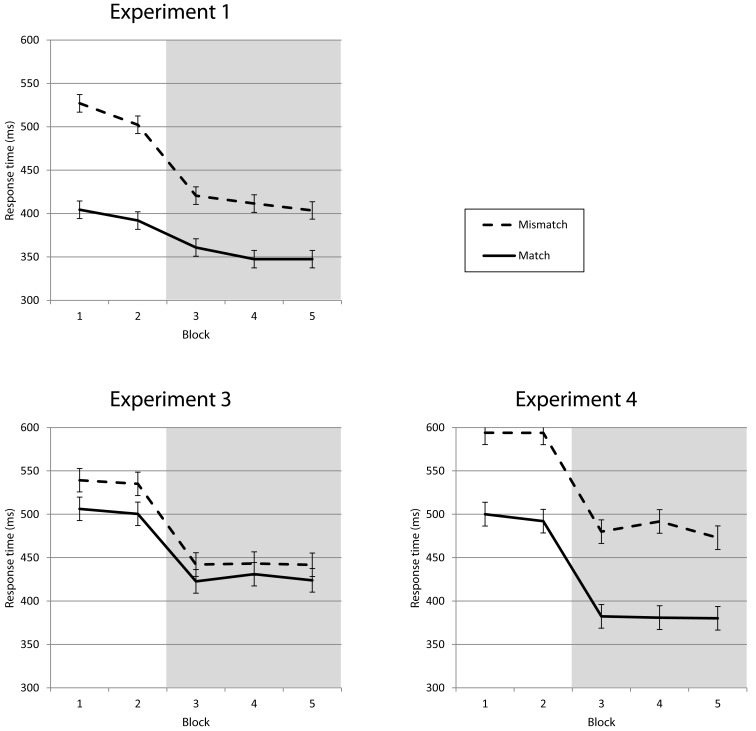
**Match vs. mismatch trials for Experiments 1, 3, and 4 (pooled over frequencies)**. Responses following correct predictions (match) in Experiment 1 are 117 ms faster on average compared to incorrect predictions (mismatch) in the first two blocks; after the introduction of the selective response deadline for the medium frequency stimulus this difference is reduced to 60 ms on average. In Experiment 3, using invalid cues (similar to the predictions of Experiment 1), the difference between match and mismatch trials averages 34 ms at the beginning and is down to 17 ms with the additional task demand. Experiment 4 shows no reduction in this mismatch effect, with 98 ms before and 100 ms after the introduction of the deadline on average.

**Figure 5:**
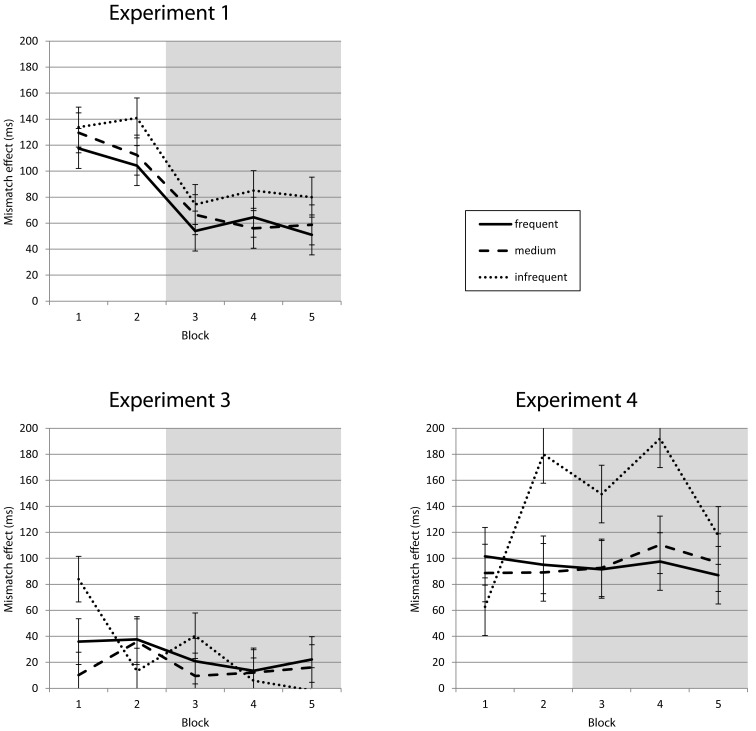
**Mismatch effect for Experiments 1, 3, and 4 (displayed by frequency)**. No differences in mismatch effect between frequencies, similar reduction (Experiment 1) or stability (Experiment 3) with the additional task goal introduced in Block 3. Values from infrequent stimuli are statistically unreliable because of the low number of match trials.

Stimuli matched predictions in 42% of all trials (with a minimum of 40% in Block 1 and a maximum of 44% in Block 4; 51% matches for the frequent stimulus, 30% for the medium, and 18% for the infrequent stimulus). Response times were shorter for trials in which the stimulus matched the participant’s prediction, as compared to mismatch trials. This match effect was visible for all stimulus frequencies. The ANOVA with the factors *match* and *frequency* revealed main effects on RT for *match*, *F*(1, 18) = 130.72, *p* < 0.001, and *frequency*, *F*(2, 36) = 74.55, *p* < 0.001, but no interaction *match* × *frequency*, *F*(2, 36) = 2.77, ns. After introducing the response deadline for the medium stimulus, the mean difference between match and mismatch trials declined from 110 ms in Block 2 to 60 ms in Block 3 (see Figures 4 and 5, top left).The ANOVA with the factors *match* and *block* revealed main effects on RT for *match*, *F*(1, 18) = 107.63, *p* < 0.001, and *block*, *F*(4, 72) = 81.14, *p* < 0.001, as well as an interaction *match* × *block*, *F*(4, 72) = 30.56, *p* < 0.001. The same effects were found in the error rates (all *p* < 0.001).

The *post hoc* estimates of stimulus occurrence in percent made by the participants also provided a good approximation of the actual frequencies, with the frequent stimulus at 63%, the medium at 24%, and the infrequent stimulus at 13%.

### Experiment 2: No predictions

One participant was excluded from analyses for producing too many false responses (>10%). Data of the remaining 21 participants were analyzed. The response deadline was on average fixed to 491 ms (SD = 89 ms), with individual participants ranging between 333 and 693 ms. On 8% of the trials with the reinforced stimulus, participants exceeded this deadline and were consequently exposed to the aversive sound (9% in Block 3, 7% in Block 4, 9% in Block 5).

The same pattern emerged as in Experiment 1: Responses were faster and more accurate to the more frequent stimuli in the first two experimental blocks, before the introduction of the response deadline. With the deadline, in the last three blocks, responses to the reinforced medium frequent stimulus became faster than responses to the frequent stimulus, while response times for all stimuli decreased (see Figure 2, top right). A two-way repeated measures ANOVA on RTs with the factors *frequency* and *block* revealed main effects of both stimulus *frequency*, *F*(2, 40) = 71.87, *p* < 0.001, and *block*, *F*(4, 80) = 58.96, *p* < 0.001, as well as an interaction, *F*(8, 160) = 25.02, *p* < 0.001. The same effects were found in the error rates (all *p* < 0.001).

The *post hoc* estimates again provided a good approximation of the actual frequencies, with the frequent stimulus at 64%, the medium at 25%, and the infrequent stimulus at 11%.

### Experiment 3: Non-informative cues

Seven participants were excluded from analyses for producing too many false responses (>10%). Data of the remaining 30 participants were analyzed. The response deadline was on average fixed at 502 ms (SD = 75 ms), with individual participants ranging between 383 and 695 ms. On 8% of the trials with the reinforced stimulus participants exceeded this deadline and were consequently exposed to the aversive sound (9% in Block 3, 7% in Block 4 and 5).

Similar to Experiment 2, RTs followed stimulus frequency in Blocks 1 and 2, but the medium frequency stimulus elicited the fastest responses when the reinforcement procedure started after Block 2 (compare Figure 2, bottom left). A two-way repeated measures ANOVA with *frequency* and *block* revealed main effects on RT for *frequency*, *F*(2, 58) = 107.33, *p* < 0.001, and *block*, *F*(4, 116) = 71.45, *p* < 0.001, as well as an interaction *frequency* × *block*, *F*(8, 232) = 61.28, *p* < 0.001. The same effects were found in the error rates (all *p* < 0.001). The RT effect of cue match was clearly present for all frequencies as well, but smaller than the effect of expectation match in Experiment 1. The ANOVA with *match* and *frequency* revealed main effects on RT for *match*, *F*(1, 29) = 21.57, *p* < 0.001, and *frequency*, *F*(2, 58) = 80.11, *p* < 0.001, but no interaction *match* × *frequency*, *F*(2, 58) = 0.43, ns. In the error rates, only frequency yielded a significant effect, *F*(2, 58) = 16.64, *p* < 0.001. After the introduction of the response deadline the match effect was diminished from 35 ms in Block 2 to 19 ms in Block 3 (see Figures 4 and 5, bottom left). The third ANOVA with *match* and *block* revealed main effects on RT for *match*, *F*(1, 29) = 23.41, *p* < 0.001, and *block*, *F*(4, 116) = 44.47, *p* < 0.001, as well as an interaction *match* × *block*, *F*(4, 116) = 13.74, *p* < 0.001. The same effects were found in the error rates (all *p* < 0.01).

The *post hoc* estimates again provided a good approximation of the actual frequencies, with the frequent stimulus at 57%, the medium at 30%, and the infrequent stimulus at 13%.

### Experiment 4: Informative cues

Four participants were excluded from analyses for producing too many false responses (>10%). Data of the remaining 18 participants were analyzed. The response deadline was on average fixed to 497 ms (SD = 120 ms), with individual participants ranging between 311 and 708 ms. On 11% of the trials with the reinforced stimulus participants passed this deadline and were consequently exposed to the aversive sound (13% in Block 3, 12% in Block 4, and 9% in Block 5).

As shown in Figure 2 (bottom right), RTs followed stimulus frequency in the first two blocks until the onset of the reinforcement of the medium frequency stimulus at the beginning of Block 3 led to faster responses to this stimulus. The two-way repeated measures ANOVA with *frequency* and *block* revealed main effects on RT for *frequency*, *F*(2, 34) = 45.83, *p* < 0.001, and *block*, *F*(4, 68) = 34.74, *p* < 0.001, as well as an interaction *frequency* × *block*, *F*(8, 136) = 22.99, *p* < 0.001. The same effects were found in the error rates (all *p* < 0.001). RT effects of match between cue and stimulus were much more pronounced than in the low validity variant explored in the previous experiment and were not reduced after the introduction of the response deadline (102 ms in Block 2, 98 ms in Block 3, see Figures 4 and 5, bottom right). Accordingly, the ANOVA with *match* and *block* revealed main effects on RT for *match*, *F*(1, 17) = 110.14, *p* < 0.001, and *block*, *F*(4, 68) = 41.30, *p* < 0.001, but no interaction *match* × *block*, *F*(4, 68) = 0.55, ns. The third ANOVA with *match* and *frequency* revealed main effects on RT for *match*, *F*(1, 17) = 113.20, *p* < 0.001, and *frequency*, *F*(2, 34) = 76.56, *p* < 0.001, but no interaction *match* × *frequency*, *F*(2, 34) = 7.56, ns.

The *post hoc* estimates again provided a good approximation of the actual frequencies, with the frequent stimulus at 56%, the medium at 30%, and the infrequent stimulus at 14%.

## Discussion

In all four experiments reported here, stimulus frequencies (60, 30, 10%) were reflected in response times and error rates, with the most frequent stimulus producing the fastest and most accurate responses. While discussion about the role of conscious intention in controlling behavior (Libet et al., 1983; Soon et al., 2008; Trevena and Miller, 2010; see Introduction) might be taken to suggest that explicit expectations merely reflect other preparatory processes but do not influence them, our results suggest that explicit expectations feed back into task processing and thus have a causal role. We disentangled explicit expectation from other forms of preparation by adding a secondary task demand. With instruction and a response deadline combined with an aversive sound, participants were encouraged to prepare for a different stimulus (i.e., the medium frequency stimulus) than the one they were expecting most often (i.e., the high frequency stimulus). Explicit expectations affected task processing even when it would have been beneficial not to rely on them: On the one hand, effects of expectation conflicted with the requirement to respond faster than the response deadline on the medium frequency stimulus. This could have largely been avoided if participants had either not have turned verbalized expectation into task preparation or, alternatively, would have started to explicitly expect the medium frequency stimulus in most or all trials. On the other hand, participants showed faster response times when their expectation matched rather than mismatched the stimulus even in case of the infrequent stimulus – which they sometimes expected. Such an expectation was mostly followed by the frequent or medium frequent rather than the infrequent stimulus. In principle one could have betted on and prepared for the frequent or medium stimulus, despite verbalizing an expectation for the infrequent one. A mismatch was much more likely than a match after such a prediction, yet matches were faster than mismatches. It would have been conceivable that participants show RT benefits of expectations matching the stimuli in case of frequent and medium frequency stimuli and a reversal of the expectation match effect in case of the infrequent (10%) stimuli. For instance, Notebaert et al. (2009) have reported that in cases with a majority of error trials RTs are prolonged after the rare correct trials rather than after error trials, suggesting that event frequency rather than match vs. mismatch of task demands and action can drive performance costs. This does not seem to count for explicit expectations, however. Thus, neither were explicit expectations themselves chosen flexibly to boost performance, nor could the aftereffects of these expectations be flexibly regulated. The results thus suggest that explicit expectations influence rather than merely reflect other preparatory processes and do so rather inflexibly. Explicit expectations seem to count – even when they are not adaptive to current task demands.

In the current experiments we took two different approaches by measuring expectations through verbal predictions and inducing them by cues. In *Experiment 1* we asked participants to verbally predict the upcoming stimulus on each trial and then respond to the actual stimulus by pressing the corresponding key. Verbal predictions (as a measure of subjective expectation) mirrored actual stimulus frequencies already in the first experimental block, with participants predicting the most frequent stimulus on a higher proportion of trials. When the imperative stimulus matched the prediction on a given trial, participants responded much faster compared to trials on which the stimulus violated their prediction. This gain was similar for all three stimulus frequencies, suggesting that participants used their predictions to prepare the response even if it was unlikely to be fulfilled (18% for the infrequent stimulus, compared to 51% for the frequent stimulus). Introducing the response deadline for the medium frequency stimulus reduced this match effect from 117 to 60 ms, while predictions themselves were not altered.

In *Experiment 2* we replicated the effects of stimulus frequency without verbal predictions, ruling out the possibility that the response time effects found in Experiment 1 were dependent on the second task of explicitly verbalizing stimulus expectations. In *Experiment 3* we induced explicit expectations through symbolic cues. As cue presentation was not related to the subsequent stimulus, their predictive value was as low as that of the self-generated predictions in Experiment 1. There was a small match effect with faster responses following correct cues (34 ms) before the introduction of the response deadline that was diminished to a statistically non-significant difference (17 ms) with the additional task demand. In *Experiment 4*, with cues correctly predicting the upcoming stimulus in 80% of all trials, there was a large match effect that was not reduced by the response deadline (98 ms before, 100 ms after the manipulation). This deviates from the patterns found in Experiments 1 and 3, where the additional task demand (fast responses on the medium frequency stimulus to avoid the aversive tone) led to a reduction in the match effect.

### Double impact of stimulus frequency

In addition to explicit expectations, RT was affected by stimulus frequency in all four experiments. This is in line with earlier calls to integrate associative as well as an expectancy-based accounts of action preparation. For instance, Miller and Anbar (1981) argue that frequency effects on response time can arise directly (through the strength of S-R associations) and indirectly (through subjective expectancies). Asking participants to verbalize their expectations (in Experiment 1) might have led to larger RT differences between stimuli of different frequency compared to the variants without predictions (Experiment 2) or with external cues (Experiments 3 and 4). Frequency effects might have been prominent on two rather than just one path in Experiment 1. As frequency effects remained evident after the introduction of the response deadline for the medium frequency stimulus, this is pointing toward an automatic effect of S-R frequency and as such toward an independent contribution of this source.

Subjective expectations measured as predictions in Experiment 1 closely mirrored the frequency pattern, a phenomenon also known as probability matching (e.g., Gaissmaier and Schooler, 2008, see below). Thus, performance in predicting the upcoming stimulus was also influenced by the given frequency pattern. The participants presumably made use of their prior experience represented in associations of varying strength. However, the *effect* of subjective expectation and the general effect of frequency on performance in the choice task appear to be independent from each other. Match effects were of similar size for all frequencies, or, to put it differently: the general effect of frequency proved to be the same, regardless of expectation match. This also holds for the experiments where cues instead of predictions were used. That is, the influence of explicit expectation on task processing appears to be different from other effects that arise from stimulus frequencies.

### Predictions: Matching vs. maximizing

Predictions were generated and used in a less than optimal manner. Participants could have maximized their correct predictions (in Experiment 1) by always predicting the most frequent stimulus (which would have lead to 60% matches). Instead, they apparently tried to reproduce the observed stimulus frequencies in their predictions (resulting in only 42% matches). This behavior is in line with the probability matching phenomenon (e.g., Gaissmaier and Schooler, 2008). Trials with expectations matching the stimulus were faster than those with a mismatch. For boosting performance in the choice reaction task it would have been favorable to choose to predict the most frequent stimulus on all trials in the first part of the experiment and the medium frequency stimulus once the response deadline on this stimulus was set in place. Maximizing has been observed in the literature on strategy change in skill acquisition (e.g., Touron and Hertzog, 2004; Gaschler and Frensch, 2007, 2009) where people tend to exclusively choose the one of two processing strategies that is the most suitable on most of the trials. This however, might be an exception as in many other task contexts probability matching has proven to be a robust phenomenon (see, e.g., Gallistel, 2005, for a discussion). He suggested that probability matching is a “hard-wired” policy which is useful in dynamic environments as it guarantees continuous sampling of the options so that an agent does not run the risk of missing to notice changes in which options are currently more or less rewarding. Our results lend further support to this “hard-wired” view, as the *influence* of the probability-matched expectations appears not to be easily adapted to more promising strategies either. However, we do not know for certain what the goals of our participants in optimizing their task performance are. It is possible that they tried to find a balance between the two tasks of realistically predicting stimuli while performing rapidly and correctly on the choice task. Therefore, instructing them to increase their proportion of matches might change the pattern of results.

### Conflicting task demand attenuates impact of expectation

The match effects we found, with faster responses following correct predictions and valid cues, are compatible with the idea that explicit expectation serves as a trigger for action preparation and thus assumes a causal role in cognitive processing. However, there are differences in the robustness of these match effects that depend on the source of expectation on the one hand and on its validity on the other hand.

The additional task demand of trying to respond quickly to the medium frequency stimulus in order to avoid hearing the unpleasant sound significantly reduced the match effects in Experiments 1 and 3, but not in Experiment 4. While in Experiment 4 the cue was highly predictive of the stimulus, explicit expectations (Experiment 1) and cues in Experiment 3 were equally unreliable. Arguably, the strong associations between cue and stimulus in Experiment 4 were still fully effective under the response deadline, whereas the impact of the unreliable predictions in the other experiments could be attenuated. Importantly, the match effect was reduced for all stimuli to a similar extent. The predictions that could have boosted the processing of the medium frequency stimulus with the deadline attached to it, were apparently not spared. Rather, participants seem to have relied somewhat less on expectations in general.

While the influence of the non-informative cues (in Experiment 3) on response time was effectively removed by the additional task demand, subjective predictions retained a significant impact. This suggests that self-generated predictions are mandatorily processed and trigger action preparation even if they are obviously unreliable and if task demands favor the preparation of a different action. As Kunde et al. (2007) argue, expectation is an integral component of action control. Expectations are always generated and translated into preparation (of perception or action) as this is usually beneficial to optimize behavior in real life. Artificial external cues do not share this processing privilege by default and have to first prove their usefulness (reliability). When they do, however, as in Experiment 4 (with 80% valid cues), they retain their influence in spite of the additional task demand.

### Expect one thing, prepare for another

The selective reinforcement of the medium frequent stimulus led to a selective speed up of responses to the reinforced stimulus. Thus, participants in our study apparently were able to predict one thing while at least partly preparing for another. A similar dissociation between explicit expectation and overt behavior has been reported before (Perruchet et al., 2006) for simple reactions in an associative learning experiment. In the “Perruchet effect,” response time (as a measure of automatic activation) decreases with increasing number of repeated associations, while explicit expectation develops in the opposite direction, increasingly favoring an alternation after longer runs of repetitions (the “gambler’s fallacy”). However, in contrast to the build-up of associative effects, in our study the change in performance occurred immediately after instructing the new requirement, rather than gradually. The abrupt effect of the deadline suggests that intentional control processes can influence the extent to which learned S-R connections impact behavior. The ordering of RTs by stimulus frequency was immediately altered. With the stimulus-specific deadline, the RT for the medium frequency stimulus surpassed RT for the frequent stimulus. In line with the intentional weighting principle proposed by Hommel et al. (2001), intentional control might put some extra strength on a response alternative that would have been otherwise weak and so alter the result of the competition for response selection. Put differently, if something we have learned earlier (as, e.g., expecting stimuli with a given frequency) conflicts with actual task goals (as, e.g., responding fast to a less expected stimulus), behavior will always be the result of resolving this – classical – conflict situation (see Botvinick et al., 2001). If expectations conflict with other task demands it seems feasible to prepare for something one is not expecting.

## Conclusion

We have shown that explicit expectation affects preparatory processes and thus assumes a causal role in controlling behavior. This finding speaks against the notion of explicit expectation as a mere by-product of preparation. When we ask participants for their subjective predictions about an upcoming event they have to respond to, they are preparing for what they say (instead of telling us what they are preparing for).

## Conflict of Interest Statement

The authors declare that the research was conducted in the absence of any commercial or financial relationships that could be construed as a potential conflict of interest.
